# Serum metabolomics and gut microbiota analysis reveal the lipid-lowering effects of fermented triple-bean soup in high-fat diet-fed mice

**DOI:** 10.3389/fnut.2025.1705483

**Published:** 2025-12-03

**Authors:** Zina Hao, Xinyi Li, Zongze Li, Menghan Ma, Yue Xi, Li Yu, Yimeng Wang, Baogang Zhang, Yunhe Xu, Lili Zhang

**Affiliations:** 1 College of Food and Health, Jinzhou Medical University, Jinzhou, China; 2 Liaoning Provincial Professional Technology Innovation Center of Meat Processing and Quality-Safety Control, Jinzhou, China; 3 College of Basic Medicine, Jinzhou Medical University, Jinzhou, China; 4 The Third Affiliated Hospital of Jinzhou Medical University, Jinzhou, China; 5 Discipline Inspection and Supervision Office, Jinzhou Medical University, Jinzhou, China; 6 College of Animal Husbandry and Veterinary Medicine, Jinzhou Medical University, Jinzhou, China

**Keywords:** metabolomics, gut microbiota, lipid-lowering, functional food, fermented triple-bean soup

## Abstract

Triple-bean soup (TBS), a traditional Chinese functional food, was innovatively fermented with lactic acid bacteria (FTBS) to enhance its hypolipidemic potential. Using a multi-omics approach integrating 16S rRNA sequencing and serum metabolomics, we systematically investigated FTBS’s effects on high-fat diet (HFD)-induced metabolic disorders in mice. FTBS significantly alleviated HFD-induced metabolic disorders, outperforming UFTBS. It remodels the microbial ecosystem by suppressing obesogenic bacteria, restoring microbial diversity and F/B balance, and increasing the abundance of *Prevotella*, *Coprococcus*, and *Oscillospira*. The levels of short-chain fatty acids (SCFAs), notably butyrate and propionate, increased by 1.8-fold following a substantial enrichment of key beneficial bacterial species, including *Prevotella* and *Coprococcus*. Metabolomic profiling identified that FTBS modulates the levels of 192 metabolites, reprogrammed key pathways, such as valine, leucine, and isoleucine biosynthesis, glycerophospholipid metabolism, citrate cycle, and primary bile acid biosynthesis. Consequently, systemic metabolomic profiles improved, manifesting as reduced hepatic steatosis and improved blood lipid levels. Our study demonstrates that FTBS ameliorates metabolic syndrome by modulating the gut-liver axis via specific microbial and metabolic shifts. These findings position FTBS as a promising nutraceutical for metabolic liver disease, merging traditional dietary knowledge with modern microbiome science.

## 1 Introduction

The global shift towards high-fat diets (HFD) has led to a significant increase in the prevalence of dyslipidemia and related chronic diseases, such as hyperlipidemia, atherosclerosis, and obesity (1). Statins, including lovastatin and simvastatin, are widely prescribed for managing hypercholesterolemia (2); however, their long-term use is associated with adverse effects, such as liver dysfunction and muscle pain (3). For individuals with early-stage hyperlipidemia, functional foods with lipid-modulating properties are increasingly recommended as a safer alternative to pharmaceutical interventions (4).

Triple-bean soup (TBS), a traditional Chinese functional food, has been used for centuries to enhance immunity and promote health. Three varieties of legumes are present in this soup: mung bean (*Vigna radiata*), rice bean (*Vigna umbellata*), and black soybean (*Glycine max*). Previous studies have demonstrated the potential of these beans as a promising source of bioactive compounds, leading to prevention and treatment of hyperlipidemia (5–10). Meanwhile, TBS has been proven to contain a variety of bioactive compounds, such as saponins, phenolic compounds, anthocyanins, and tannins (11). These bioactive components make TBS a promising candidate for the development of functional foods aimed at managing hyperlipidemia (6, 12).

Fermentation, a traditional food preservation method, has gained attention as a biotransformation strategy to enhance the functional properties of foods (13, 14). Fermentation not only increases the diversity and concentration of bioactive compounds in plant-based foods but also enhances their prebiotic potential, which can positively influence metabolic health (15, 16). For instance, fermented black tartary buckwheat by *Bacillus* sp. DU-106 has been shown to improve gut microbiota composition and reduce blood lipid levels in rats (17). Similarly, fermented ougan juice, enriched with flavonoids, alleviates obesity and hyperlipidemia in HFD-fed mice (18). These findings highlight the potential of fermented foods to modulate lipid metabolism through gut microbiota and metabolic pathways (19, 20). Probiotics are live microorganisms that, when administered in adequate amounts, confer a health benefit on the host (21). Probiotics (e.g., *Levilactobacillus brevis* FZU0713 and *Pediococcus acidilactici* FZU106) and their metabolites can improve blood lipid levels, which works synergistically with the effects of the bioactive compounds (19, 22).

In preliminary experiments, we found that TBS fermented with lactic acid bacteria (FTBS) strongly inhibited cholate *in vitro*, which indicated that FTBS had the potential to lower blood lipids. Therefore, this study aimed to investigate the hypolipidemic effects of FTBS in HFD-fed mice and elucidate its regulatory mechanisms through comprehensive analysis of gut microbiota and serum metabolomics.

## 2 Materials and methods

### 2.1 Bacterial strains and fermentation

The TBS solution was prepared according to the traditional method. Equal parts of legumes (100 g each of mung beans, red beans, and black beans) were mixed with boiling water at a ratio of 1:8 (m/v). Simultaneously, 6% (m/v) brown sugar and 0.5% (m/v) glucose were added. The mixture was boiled at 100 °C for 18 min. The solid residues were then removed using a 120-mesh sieve. The resulting filtrate was bottled and subjected to pasteurization at 95 °C for 15 min. The entire sample preparation process strictly adhered to aseptic techniques, with immediate filling and sealing upon completion.

*Lactococcus lactis* subsp. *lactis* YM313 and *Lactobacillus casei* YQ336 were isolated from Beijing Douzhi, a traditional Chinese fermented beverage. *Lactococcus lactis* subsp. *lactis* YM313 was cultured for two generations (15 h per generation) in TBS medium containing 2% (m/v) glucose and 1.5% (m/v) sodium β-glycerophosphate. *Lactobacillus casei* YQ336 was cultured for two generations (15 h per generation) in TBS medium containing 2% (m/v) glucose. Sterilized TBS (1 kg) was fermented with a mixed starter culture of *Lactococcus lactis* subsp. *lactis* YM313 and *Lactobacillus casei* YQ336 (inoculum size 6%, YM313:YQ336 = 1:1). The fermentation was carried out at 30 °C for 15 h, and then stored sealed at 4 °C. Unfermented triple-bean soup (UFTBS) was also stored under the same conditions in an airtight container.

### 2.2 Bioactive components determination

The determination of total flavonoids and total phenols was carried out using the aluminum chloride colorimetric method and the Folin–Ciocalteu method, respectively (23, 24). The organic acids were determined by HPLC (Agilent, United States) (25).

### 2.3 Experimental animals and experimental design

Male Kunming mice (5-week, 20–25 g), provided by the Experimental Animal Center of Jinzhou Medical University (Jinzhou, China, Certificate Number SYXK(LIAO) 2019-0007), were housed in a pathogen-free facility (25 ± 3 °C, 55 ± 5% relative humidity, and a 12/12-h light/dark cycle). All animal procedures conducted in this study were approved by the Animal Laboratory Ethics Committee of the Jinzhou Medical University (Permit Number: 20230416).

After 1 week of adaptation, the mice were randomly divided into four groups: mice fed with a normal diet (NFD, *n* = 10), mice fed with an HFD (HFD, *n* = 10), mice fed with an HFD supplemented with UFTBS (UFTBS, *n* = 10), mice fed with an HFD supplemented with FTBS (FTBS, *n* = 10). The HFD contained 49% basal feed, 1.5% cholesterol, 0.5% sodium cholate, 10% lard, 20% fructose, 2% multivitamins, 2% calcium bicarbonate, and 12% casein (1140A, Liaoning Changsheng biotechnology Co., Ltd., Liaoning, China). The specific grouping information is presented in Supplementary Table S1. Body weight and food and water intake were recorded weekly throughout the 12-week trial.

### 2.4 Sample collection

After euthanasia, mouse livers were harvested, rinsed with cold sodium chloride solution (0.9%, w/w), and weighed. A portion of each liver was fixed in 10% formalin, while the remaining tissues, along fresh fecal pellets, were stored at −80 °C. Serum samples were collected by centrifuging the blood samples at 3,500 × g for 10 min.

### 2.5 Biochemical parameter analyses

Total cholesterol (TC), triglyceride (TG), high-density lipoprotein cholesterol (HDL-C), low-density lipoprotein cholesterol (LDL-C), catalase (CAT), malondialdehyde (MDA), superoxide dismutase (SOD), and glutathione peroxidase (GSH-Px) were quantified using respective kits (Jiancheng Bioengineering Co., Nanjing, China).

### 2.6 Histological analysis

Liver tissues fixed in 10% formalin for 48 h were dehydrated using a graded alcohol series and embedded in paraffin. Liver sections (4-μm thick) were stained with hematoxylin–eosin (H&E) and observed under a light microscope (Leica, Germany) at 200× magnification (26).

### 2.7 Gut microbiota analysis

The fecal genomic DNA extraction, PCR amplification, and sequencing work were completed by Majorbio (Shanghai, China) and followed the previously described experimental procedures (27). Metagenomic DNA was extracted using a Genomic DNA Kit (Axygen Scientific, Inc., Union City, CA, United States). The total extracted microbial DNA was analyzed using agarose gel electrophoresis. The V3–V4 hypervariable regions of the 16S rRNA gene were amplified and the PCR products were analyzed using agarose gel electrophoresis, purified a Gel Extraction Kit (Axygen Scientific Inc., Union, CA, United States) and quantified using a QuantiFluor^™^-ST Fluorometer (Promega Biotech Co., Beijing, China). An Illumina MiSeq platform (Shanghai MajorbioBio-pharmTechnology Co., Ltd., Shanghai, China) was used for sequencing and analysis. Ambiguous and low-quality reads were excluded from sequencing results. QIIME2 with DADA2 analysis was used to denoise data. Redundancy was then eliminated to obtain features. α-diversity (Chao1, Observed species, Shannon and Simpson) and Venn diagrams were created using QIIME2 (2019.4), and the results were visualized using R software (V2.15.3). A principal component analysis (PCA) score plot was constructed using R software based on the relative abundance of gut microbiota at the genus level. Taxonomic composition analysis using QIIME2 was performed to identify differences at the phylum level among the groups. The raw sequencing data have been deposited in the NCBI BioProject database under accession number PRJNA1356646.

### 2.8 Analysis of short chain fatty acids

The extraction and analysis of SCFAs were carried out using a previously described method, with modifications (28). Briefly, mouse feces (0.2 g) were mixed with 1.0 mL of 0.01% H_2_SO_4_, treated with ultrasound in ice water bath for 10 min and centrifuged (10,000 × g, 4 °C, 20 min). The supernatant was filtered through a 0.45 μm membrane and then analyzed by gas chromatography (7890A, Agilent Technologies, Santa Clara, United States) using a KB-1701 column (30 m × 0.32 mm × 0.25 μm) and an FID detector. The inlet temperature was 250 °C and the shunt ratio was 1:1. Nitrogen was used as the carrier gas at a flow rate of 2.0 mL/min. The hydrogen flow rate was 40 mL/min and airflow rate was 450 mL/min. The initial oven temperature was 90 °C, which was maintained for 5 min, and then increased to 250 °C at the rate of 20 °C/min. The results of SCFAs (acetic acid, propionic acid, butyric acid, isobutyric acid, valeric acid, and isovaleric acid) were expressed in μg/g of feces.

### 2.9 Nontargeted metabolomics analysis

#### 2.9.1 Sample preparation

Serum samples (50 μL) were mixed with 150 μL methanol-acetonitrile solution (1:1, v:v), and extracted by ultrasound for 30 min (4 °C). The samples were incubated at 20 °C for 30 min and centrifuged for 20 min (12,000 × g, 4 °C). The supernatant was evaporated to dryness under nitrogen, and 100 μL of acetonitrile-aqueous solution (1:1, v:v) was added for resolution. Then, the samples were extracted by ultrasound for 5 min (4 °C) and centrifuged for 15 min (12,000 × g, 4 °C). The supernatant was collected for ultra-performance liquid chromatography-quadrupole time-of-flight mass spectrometry (UPLC-Q-TOF/MS). Quality control samples were prepared from each sample.

#### 2.9.2 UPLC-Q-TOF/MS analysis

The detection of serum metabolites was performed using a UHPLC-Exploris240 system (Thermo Fisher Scientific, Waltham, MA, United States). Chromatographic conditions were as follows: chromatographic column used was ACQUITY UPLC HSS T3 (100 mm × 2.1 mm i.d., 1.8 μm; Waters, Milford, United States); mobile phase A consisted of 95% water + 5% acetonitrile; mobile phase B consisted of 47.5% acetonitrile + 47.5% isopropyl alcohol + 5% water; sample size was 3 μL; and column temperature was 40 °C. The samples were ionized using electrospray to acquire mass spectra, and the signals were collected in both positive and negative ion scanning modes. The detailed parameters are listed in Supplementary Table S2.

#### 2.9.3 Data processing and analysis

Raw data were imported into the metabolomic processing software Progenesis QI (Waters Corporation, Milford, United States) for baseline filtering, peak identification, integration, retention time correction, and peak alignment. This software was used to identify the characteristic peaks. MS and MS/MS data were matched with the metabolic database,^1^ and the mass error threshold was set as <10 ppm. Metabolites were identified according to the secondary mass spectral matching score.

The data matrix obtained from the database search was uploaded to the Majorbio Cloud Platform^2^ for analysis. Data preprocessing was performed as follows: the 80% rule was applied to remove missing values, retaining variables with non-zero values in at least 80% of the samples within any one group. The remaining missing values were then imputed using the minimum value found in the original matrix. To reduce errors from sample preparation and instrument instability, the sum normalization method was applied to normalize the response intensity of the mass spectral peaks across all samples, resulting in a normalized data matrix. Furthermore, variables with a relative standard deviation (RSD) >30% in the quality control (QC) samples were removed. Finally, a log10 transformation was applied to yield the final data matrix for subsequent analysis.

Subsequently, the preprocessed data matrix was subjected to both principal component analysis (PCA) and orthogonal projections to latent structures-discriminant analysis (OPLS-DA) using the ropls package (Version 1.6.2) in R. The stability of the OPLS-DA model was assessed with a 7-fold cross-validation. Significantly altered metabolites were identified based on the variable importance in projection (VIP) values obtained from the OPLS-DA model and the *p*-values from Student’s *t*-test. Metabolites with a VIP >1 and a *p*-value <0.05 were defined as statistically significant.

The differentially abundant metabolites were annotated against the KEGG database^3^ to identify the metabolic pathways they are involved in. Pathway enrichment analysis was then performed using the Python package scipy.stats. The Fisher’s exact test was employed to identify the biological pathways most significantly associated with the experimental treatment.

### 2.10 Statistical analysis

Data are presented as the means *±* standard deviation. One-way analysis of variance (ANOVA), followed by Duncan’s and Tukey’s tests for multiple comparisons were used to determine statistical significance using SPSS (v17.0, SPSS Inc., Chicago, IL, United States). A *p* < 0.05 was considered statistically significant.

## 3 Results

### 3.1 Analysis of bioactive components

Table 1 shows the total flavonoids and total phenols content of FTBS was significantly higher than that of UFTBS after fermentation, (*p* < 0.05). The content of lactic acid in organic acids has sharply increased, while the content of acetic acid has risen (*p* < 0.05). However, the contents of citric acid, tartaric acid and oxalic acid have decreased (*p* < 0.05).

**Table 1:** Bioactive components of UFTBS and FTBS.

Component	UFTBS	FTBS
Total flavonoids (mg/mL)	3.43 ± 0.15^b^	4.51 ± 0.21^a^
Total phenols (mg/mL)	0.99 ± 0.02^b^	1.12 ± 0.02^a^
Organic acid (μg/mL)		
Lactic acid	0.55 ± 0.07^b^	661.72 ± 4.90^a^
Acetic acid	266.51 ± 4.32^b^	305.97 ± 4.69^a^
Citric acid	301.82 ± 6.12^a^	288.60 ± 4.28^b^
Tartaric acid	211.94 ± 4.67^a^	199.60 ± 5.39^b^
Oxalic acid	369.97 ± 5.09^a^	269.16 ± 4.30^b^
Total	1150.79 ± 20.27^b^	1725.05 ± 23.56^a^

All data presented as means ± SD. Data with different letters are significantly different (*p* < 0.05).

### 3.2 FTBS improves lipid metabolism and liver health in HFD-fed mice

**Figure 1:**
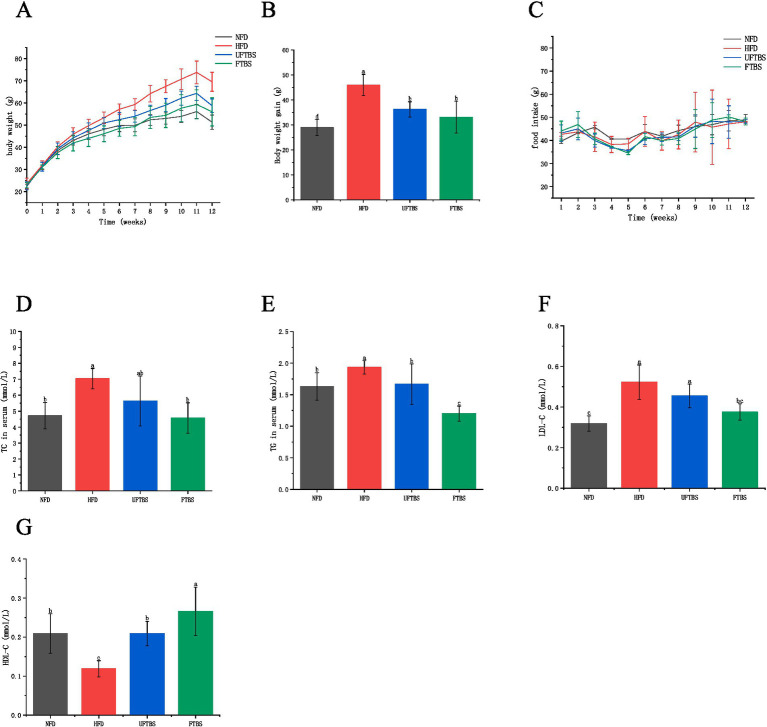
Effects of FTBS on body weight **(A)**, body weight gain **(B)**, food intake **(C)**, serum TC **(D)**, TG **(E)**, LDL-C **(F)**, and HDL-C **(G)** in mice.

During the experimental period, body weight increased gradually in all groups, with the highest weight gain observed in the HFD group. After 12 weeks, the body weight of HFD-fed mice was significantly higher than that of the NFD group (*p* < 0.05) (Figure 1A). (Since the growth rate of the mice was not high in the later stage of the experiment, and it was the weight of the mice measured after they had fasted for 24 h. Therefore, the body weight of the mice at week 12 decreased compared to that at week 11.) Both FTBS and UFTBS significantly attenuated HFD-induced weight gain (*p* < 0.05) (Figure 1B). Specifically, FTBS and UFTBS reduced body weight by 28.03 and 21.30%, respectively, compared to the HFD group. No significant differences in food intake were observed among the groups (*p* > 0.05) (Figure 1C), indicating that the weight-reducing effects of FTBS and UFTBS were not due to changes in food consumption.

Serum lipid analysis revealed that HFD significantly elevated TC, TG, and LDL-C levels while reducing HDL-C levels compared to the NFD group (*p* < 0.05). However, supplementation with FTBS and UFTBS effectively reversed these HFD-induced alterations. Notably, FTBS demonstrated superior efficacy over UFTBS in reducing TC, TG, and LDL-C levels and increasing HDL-C levels (Figures 1D–G).

All data presented as means ± SD. Data with different letters are significantly different (*p* < 0.05).

**Figure 2:**
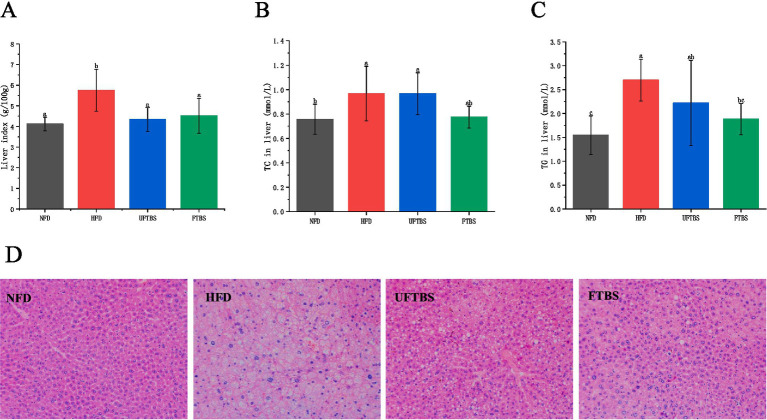
Effects of FTBS on liver index **(A)**, liver TC level **(B)**, liver TG level **(C)**, and histopathology **(D)** (magnification: 200×). All data presented as means ± SD. Data with different letters are significantly different (*p* < 0.05).

Following intervention with both UFTBS and FTBS, liver indices were significantly reduced to levels comparable to those in the NFD group (Figure 2A). Compared to the NFD group, HFD feeding markedly increased hepatic TG and TC levels (*p* < 0.05). However, FTBS supplementation effectively prevented the excessive accumulation of hepatic TG and TC (*p* < 0.05) (Figures 2B,C), demonstrating its superior lipid-lowering efficacy compared to UFTBS.

Histopathological analysis of liver tissues stained with hematoxylin and eosin (H&E) revealed significant lipid droplet accumulation, hepatocyte enlargement, and cytoplasmic pallor in HFD-fed mice (Figure 2D). While UFTBS intervention partially restored hepatocyte size, residual lipid droplets were still observed. In contrast, FTBS treatment not only reduced lipid droplet accumulation but also significantly improved hepatocyte morphology, indicating a more pronounced protective effect against HFD-induced hepatic steatosis.

### 3.3 Effect of FTBS on antioxidant activity in HFD-fed mice

**Figure 3:**
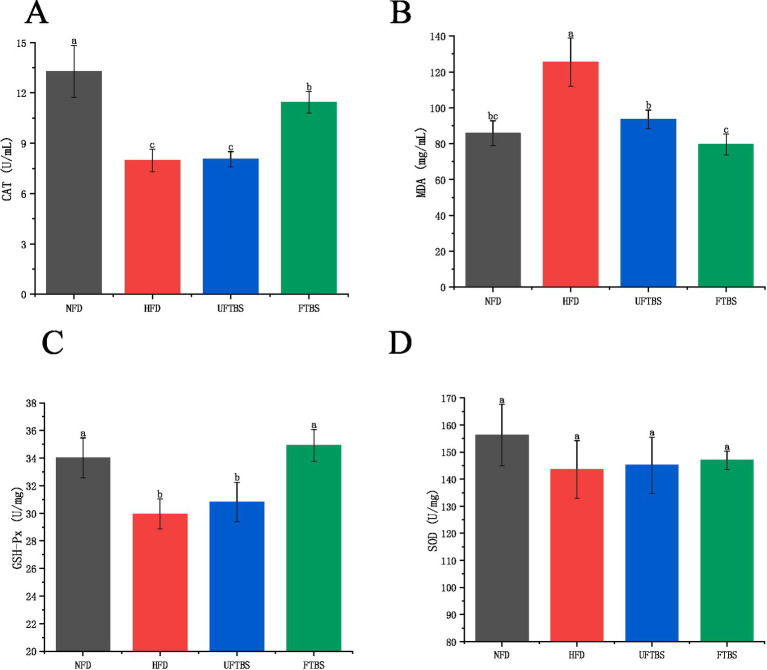
Effects of FTBS on serum CAT **(A)**, serum MDA **(B)**, liver GSH-Px **(C)**, and liver SOD **(D)** in mice. All data presented as means ± SD. Data with different letters are significantly different (*p* < 0.05).

To evaluate the antioxidant effects of FTBS, we measured the levels of serum CAT, MDA, liver GSH-Px, and SOD. HFD feeding significantly induced oxidative stress, as evidenced by elevated MDA levels and reduced CAT and GSH-Px activities compared to the NFD group (*p* < 0.05). However, supplementation with FTBS effectively mitigated these oxidative stress markers, significantly improving CAT and GSH-Px levels and reducing MDA levels (*p* < 0.05) (Figures 3A–D). In contrast, UFTBS only significantly reduced MDA levels (*p* < 0.05), with no notable improvements in other antioxidant indicators compared to the HFD group (*p* > 0.05). These results suggest that FTBS, but not UFTBS, possesses potent antioxidant properties that counteract HFD-induced oxidative damage.

### 3.4 FTBS modulates gut microbiota composition and enhances SCFA production in HFD-fed mice

**Figure 4:**
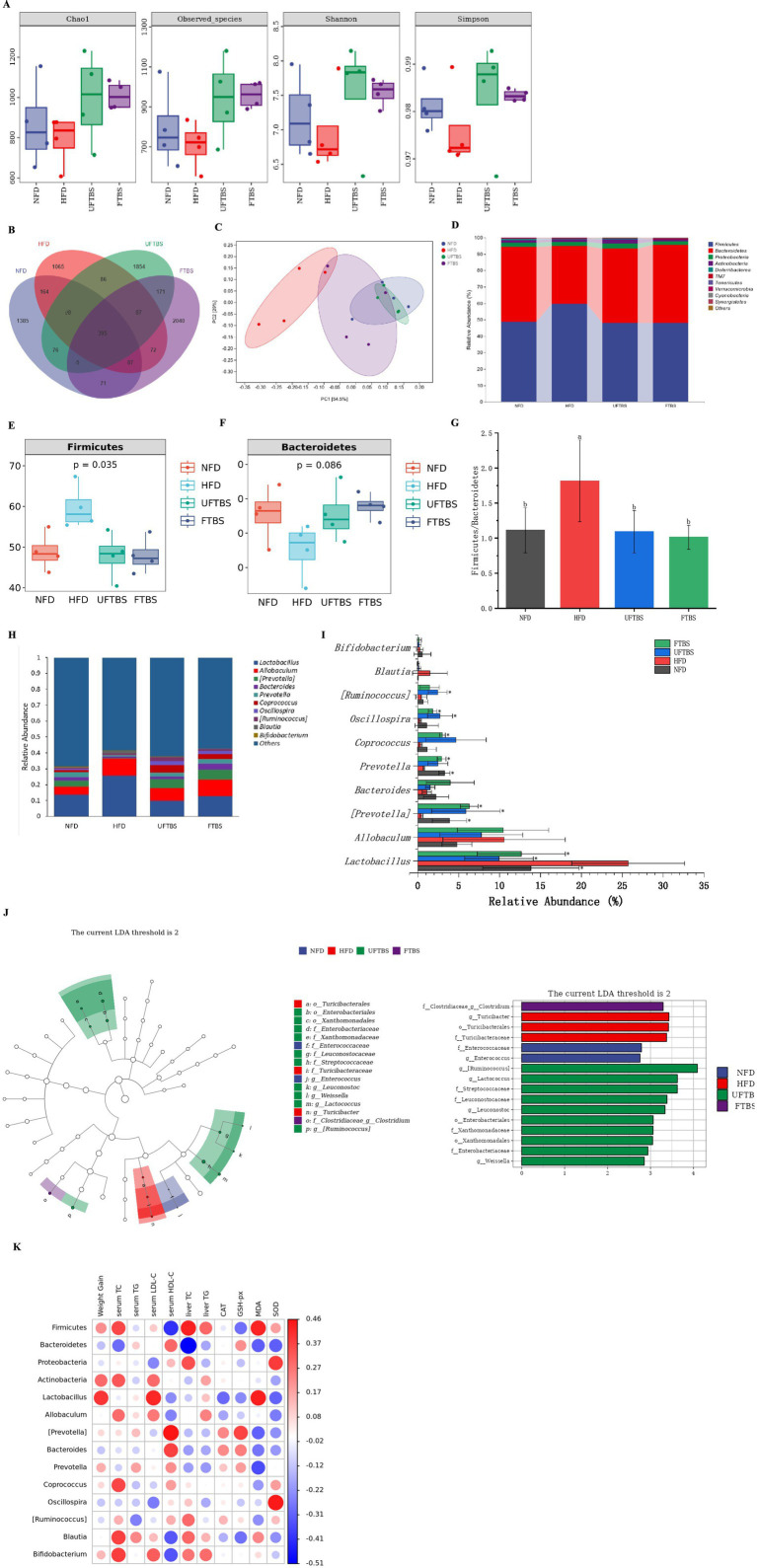
Effects of FTBS on α-diversity (Chao1 index, Observed species, Shannon index and Simpson index) **(A)**, Venn diagram **(B)**, PCA **(C)**, the relative abundance at levels of phylum (top10) **(D)**, the relative abundance of Firmicutes **(E)**, the relative abundance of Bacteroidetes **(F)**, the ratio of Firmicutes/Bacteroidetes **(G)**, the relative abundance at levels of genus (top10) **(H)**, the significantly changed bacteria at the genus level (top10) **(I)**, identification of most characteristic taxa among NFD, HFD, UFTBS, and FTBS groups by linear discriminant analysis (LDA) effect size (LEfSe) **(J)**, and spearman’s correlation between gut microbial phylotypes and host phenotypes **(K)**. All data presented as means ± SD. Data with different letters are significantly different (*p* < 0.05). ^*^p < 0.05 versus the HFD group.

Both FTBS and UFTBS interventions prevented the reduction in gut microbiota richness (Chao1 and Observed species indices) and diversity (Shannon and Simpson indices) induced by a HFD (Figure 4A). HFD significantly decreased the operational taxonomic unit (OTU) number of the gut microbiota, whereas FTBS and UFTBS supplementation increased OTU abundance, with FTBS showing a more pronounced effect than UFTBS (Figure 4B). Principal component analysis (PCA) revealed distinct clustering of gut microbiota compositions among the NFD, HFD, FTBS, and UFTBS groups. Notably, the microbiota profiles of FTBS- and UFTBS-treated mice were more closely aligned with the NFD group than with the HFD group, indicating partial restoration of gut microbiota homeostasis (Figure 4C).

At the phylum level, Firmicutes and Bacteroidetes were the dominant taxa (Figure 4D). HFD feeding increased the abundance of Firmicutes while reducing Bacteroidetes, resulting in an elevated F/B ratio from 1.11 to 1.82. Both FTBS and UFTBS interventions reversed this trend, with FTBS exhibiting a more significant reduction in the F/B ratio compared to UFTBS (Figures 4E–G).

At the genus level, FTBS and UFTBS increased the relative abundances of beneficial bacteria such as *Prevotella*, *Coprococcus*, *Oscillospira*, and *Bacteroides*, while reducing the abundances of *Lactobacillus* and *Blautia* in HFD-fed mice (Figures 4H,I). Linear discriminant analysis effect size (LEfSe) analysis identified 16 differentially abundant taxa across the four groups, including seven genera. The NFD group was characterized by a predominance of *Enterococcus*, while the HFD group was enriched with *Turicibacter*. The UFTBS group was dominated by *Ruminococcus*, *Lactococcus*, *Leuconostoc*, and *Weissella*, whereas the FTBS group showed a higher abundance of *Clostridium*. These findings suggest that specific microbial taxa may serve as potential indicators for the amelioration of HFD-induced hyperlipidemia (Figure 4).

Spearman’s correlation analysis further elucidated the relationships between gut microbiota and host phenotypes. Firmicutes abundance was positively correlated with TC, TG, and MDA levels, and negatively correlated with HDL-C and GSH-Px levels. In contrast, Bacteroidetes abundance was negatively correlated with TC levels. *Lactobacillus* was positively associated with weight gain, LDL-C, and MDA levels, while *Prevotella* showed positive correlations with HDL-C and GSH-Px levels. *Bacteroides* was positively correlated with HDL-C levels, and *Oscillospira* was positively associated with SOD levels. Additionally, *Blautia* and *Bifidobacterium* exhibited positive correlations with TC levels (Figure 4K).

**Figure 5:**
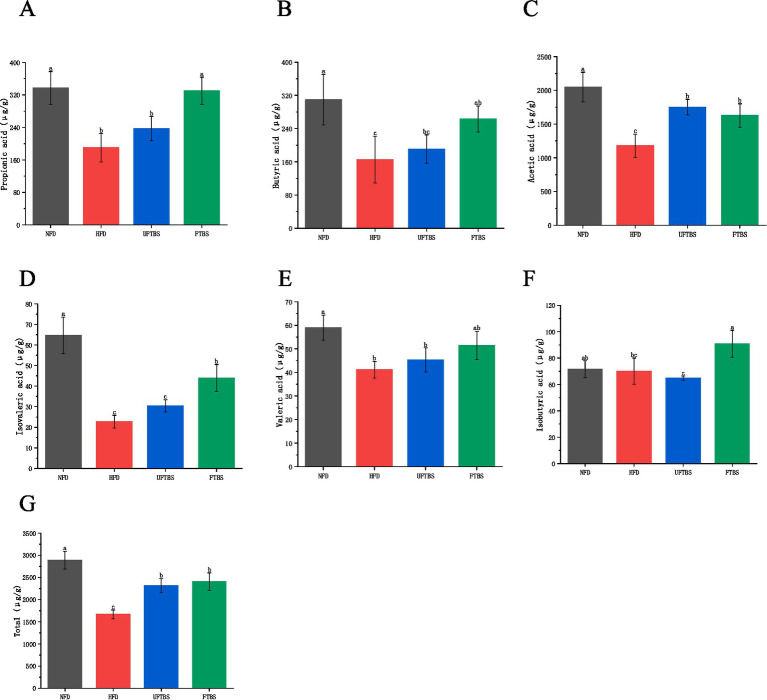
Effects of FTBS on the contents of total SCFAs **(A)**, acetic acid **(B)**, propionic acid **(C)**, butyric acid **(D)**, isobutyric acid **(E)**, valeric acid **(F)**, and isovaleric acid **(G)**. All data presented as means ± SD. Data with different letters are significantly different (*p* < 0.05).

The levels of short-chain fatty acids (SCFAs) in fecal samples were quantified, with acetic acid, propionic acid, and butyric acid being the predominant SCFAs across all groups (Figure 5). Compared to the normal diet (NFD) group, HFD feeding significantly reduced SCFA levels (*p* < 0.05). However, supplementation with FTBS markedly increased the concentrations of acetic acid, propionic acid, butyric acid, isobutyric acid, and isovaleric acid (*p* < 0.05). In contrast, UFTBS intervention only significantly elevated acetic acid levels (*p* < 0.05). Total SCFA concentrations increased by 44.14 and 39.04% in the FTBS and UFTBS groups, respectively, compared to the HFD group, with FTBS showing a more pronounced effect (Figure 5).

### 3.5 FTBS modulates serum metabolomic profiles in HFD-fed mice

**Figure 6:**
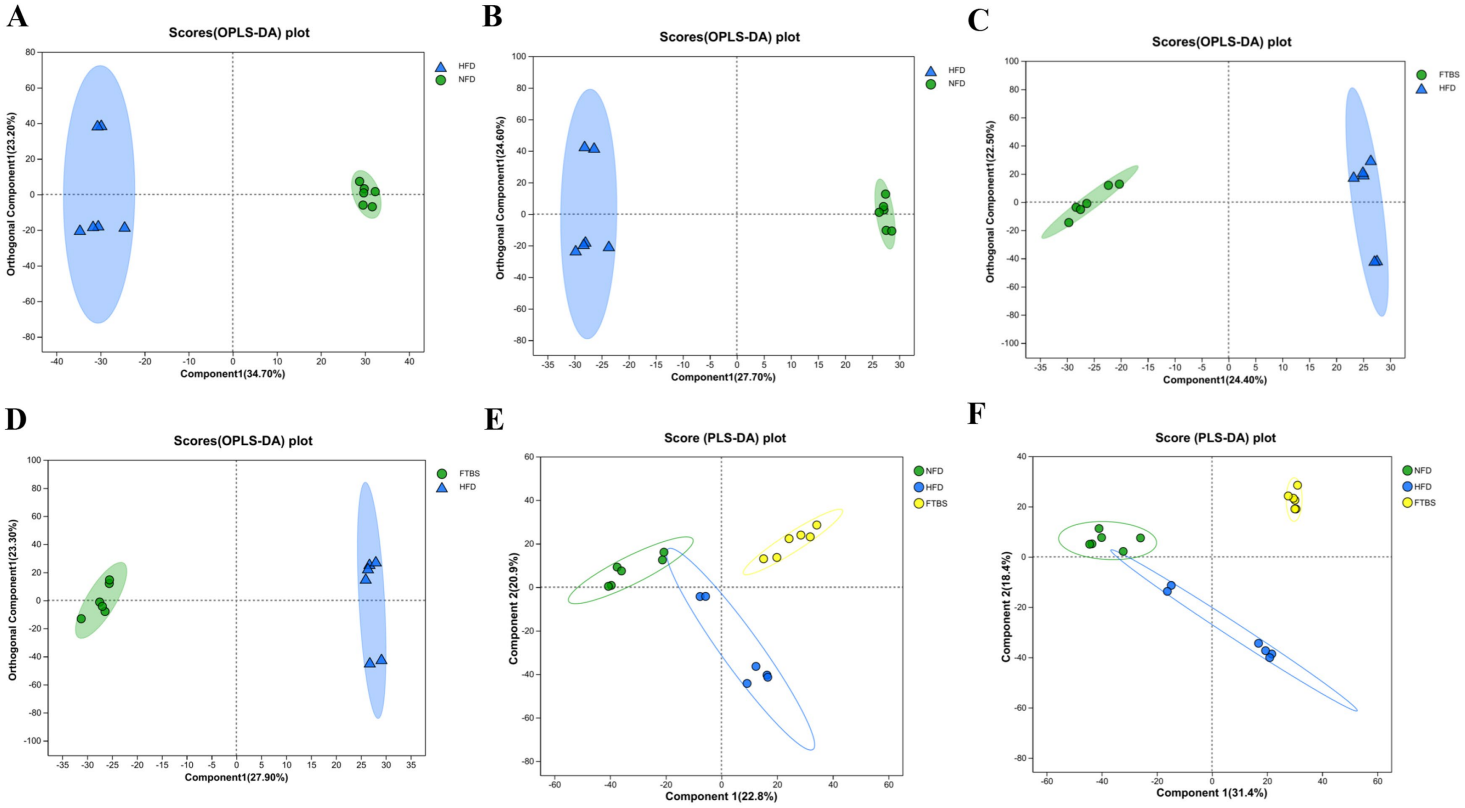
OPLS-DA score plots between NFD and HFD groups in positive (**A**, *R*^2^ = 0.813, *Q*^2^ = 0.66) and negative (**B**, *R*^2^ = 0.8, *Q*^2^ = 0.702) mode; OPLS-DA score plots between HFD and FTBS groups in positive (**C**, *R*^2^ = 0.8475, *Q*^2^ = 0.703) and negative (**D**, *R*^2^ = 0.986, *Q*^2^ = 0.865) mode; PLS-DA score plots for the NFD, HFD, UFTBS and FTBS groups in positive **(E)** and negative **(F)** mode.

Non-targeted metabolomics was employed to analyze serum metabolites in the NFD, HFD, and FTBS groups. Orthogonal partial least squares-discriminant analysis (OPLS-DA) and partial least squares-discriminant analysis (PLS-DA) score plots revealed significant differences in serum metabolic profiles between the NFD and HFD groups (Figures 6A,B). Following FTBS treatment, a clear separation was observed between the HFD and FTBS groups (Figures 6C,D). PLS-DA score plots further demonstrated that the serum metabolic profiles of FTBS-treated mice were more closely aligned with those of the NFD group than with the HFD group (Figures 6E,F).

**Figure 7:**
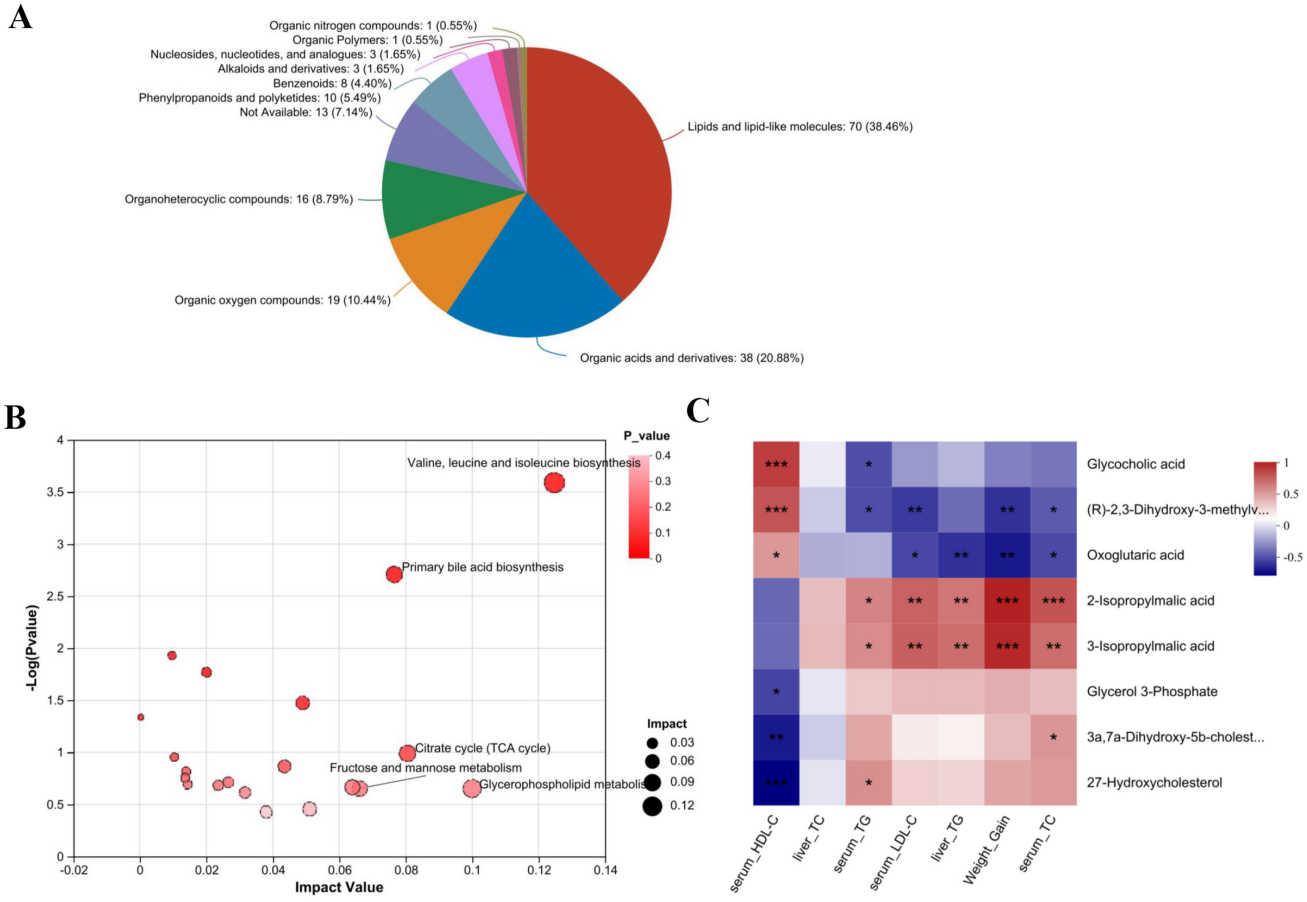
Classification of 192 differential metabolites by HMDB **(A)**; KEGG topology analysis of 192 differential metabolites **(B)**; Correlations of eight metabolites with biochemical parameters **(C)**.

Based on UPLC-QTOF/MS data, 723 differentially expressed metabolites were identified between the NFD and HFD groups (*p* < 0.05, VIP >1). Among these, FTBS intervention restored 192 metabolites to near-normal levels (Supplementary Figure S1). The specific information of these metabolites is provided in Supplementary Table S3. These metabolites were classified using the Human Metabolome Database (HMDB), with lipids and lipid-like molecules representing the largest proportion (38.46%), followed by organic acids and derivatives (20.88%), organic oxygen compounds (10.44%), and organoheterocyclic compounds (8.76%) (Figure 7A). Metabolic pathway enrichment analysis via MetaboAnalyst revealed that the differentially regulated metabolites were primarily involved in valine, leucine, and isoleucine biosynthesis; glycerophospholipid metabolism; citrate cycle (TCA cycle); and primary bile acid biosynthesis (Figure 7B). Among these metabolites, eight are identified as significant and are enriched in these pathways (Table 2). The correlations between these eight metabolites and biochemical parameters are shown in Figure 7C. The results indicate that the altered metabolites in valine, leucine, and isoleucine biosynthesis ((R)-2,3-Dihydroxy-3-methylvalerate, 2-Isopropylmalic acid, 3-Isopropylmalic acid), primary bile acid biosynthesis (Glycocholic acid, 3a,7a-Dihydroxy-5b-cholestane, 27-Hydroxycholesterol), citrate cycle (Oxoglutaric acid) and glycerophospholipid metabolism (Glycerol 3-Phosphate) show significant associations with blood lipid levels (Figure 7C).

**Table 2:** Potential biomarkers and the related pathways.

Metabolite	Adducts	Formula	HMDB class	*M*/*Z*	Related pathway	Level
(R)-2,3-Dihydroxy-3-methylvalerate	M + ACN + H	C_6_H_12_O_4_	Fatty acyls	190.1073	Valine, leucine, and isoleucine biosynthesis	↑
Oxoglutaric acid	M − H	C_5_H_6_O_5_	Keto acids and derivatives	145.0143	Citrate cycle	↑
2-Isopropylmalic acid	M + FA − H	C_7_H_12_O_5_	Fatty acyls	221.0666	Valine, leucine, and isoleucine biosynthesis	↓
3-Isopropylmalic acid	M + FA − H	C_7_H_12_O_5_	Fatty acyls	221.0667	Valine, leucine, and isoleucine biosynthesis	↓
Glycocholic acid	M + Cl	C_26_H_43_NO_6_	Steroids and steroid derivatives	500.278	Primary bile acid biosynthesis	↑
3a,7a-Dihydroxy-5b-cholestane	M + FA − H	C_27_H_48_O_2_	Steroids and steroid derivatives	449.3634	Primary bile acid biosynthesis	↓
27-Hydroxycholesterol	M + FA − H	C_27_H_46_O_2_	Steroids and steroid derivatives	447.3472	Primary bile acid biosynthesis	↓
Glycerol 3-phosphate	M − H	C_3_H_9_O_6_P	Glycerophospholipids	171.0064	Glycerophospholipid metabolism	↓

## 4 Discussion

### 4.1 FTBS improves lipid metabolism and antioxidant activity

TBS, a traditional Chinese functional food, has been used for centuries for its antioxidant, hypolipidemic, and hypoglycemic properties. Fermentation enhances the bioactive compounds in TBS, further boosting its lipid-lowering potential. The gut microbiota plays a pivotal role in lipid metabolism, and modulating its composition can alleviate hyperlipidemia (29). Metabolites, as regulators of host phenotypes, further underscore the importance of understanding the interplay between gut microbiota and metabolic health (28). This study demonstrates that FTBS ameliorates HFD-induced hyperlipidemia by regulating gut microbiota and serum metabolic profiles.

Epidemiological studies highlight that elevated LDL-C and TG levels are significant risk factors for cardiovascular diseases, particularly atherosclerotic cardiovascular disease (ASCVD) (20, 28). Consistent with previous findings, HFD feeding in this study led to increased body weight, serum TC, TG, and LDL-C levels, along with decreased HDL-C levels (30, 31). FTBS and UFTBS interventions reversed these changes, with FTBS showing superior efficacy in reducing hepatic lipid accumulation and improving dyslipidemia. These results suggest that fermentation enhances the lipid-regulating properties of TBS.

FTBS also significantly increased antioxidant activity in serum and liver tissues, likely due to the release of polyphenols and flavonoids during lactic acid bacteria fermentation (32). Dietary polyphenols and flavonoids are known to mitigate oxidative stress by scavenging reactive oxygen species (ROS) and preventing ROS-related pathologies (33). For instance, citrus polyphenols have been shown to regulate blood lipids by enhancing SOD and GSH activities while reducing MDA levels (34). Additionally, lactic acid bacteria themselves exhibit antioxidant properties, further contributing to lipid metabolism regulation (35).

### 4.2 FTBS modulates gut microbiota and enhances SCFA-mediated metabolic pathways

#### 4.2.1 Modulation of gut microbiota composition and function

The gut microbiota is increasingly recognized as a therapeutic target for dyslipidemia and other metabolic disorders (36, 37). A diverse gut microbiota is essential for metabolic health (38). In this study, HFD significantly reduced gut microbiota diversity, consistent with previous findings (39, 40). Reduced microbial diversity in hyperlipidemia and obesity is often associated with the loss of symbiotic bacteria, leading to impaired nutrient absorption and metabolism, which can exacerbate chronic diseases (41). FTBS and UFTBS treatments reversed the reduction in intestinal microbial diversity induced by the HFD. The Venn diagram confirmed that FTBS had richer diversity at the OTU level than UFTBS.

Firmicutes and Bacteroidetes are the dominant phyla in the gut microbiota, and their abundance is closely linked to obesity and metabolic health (41). Firmicutes abundance positively correlates with lipid droplet accumulation, which promotes fatty acid absorption and contributing to obesity and atherosclerosis (38). Bacteroidetes, known for their role in glucose and lipid metabolism, were positively correlated with improved metabolic outcomes (31). HFD increased Firmicutes abundance while reducing Bacteroidetes, leading to an elevated F/B ratio. Increased F/B ratio enhances food energy absorption, and easily induces obesity, leading to metabolic disorders (42). FTBS and UFTBS interventions reversed this trend, with FTBS showing a more pronounced reduction in the F/B ratio.

At the genus level, FTBS increased the abundance of beneficial bacteria such as *Prevotella*, *Coprococcus*, and *Oscillospira*, while reducing *Lactobacillus* and *Blautia*. *Prevotella*, a polysaccharide-digesting bacterium, stimulates the AMPK signaling pathway, alleviating glucose and lipid metabolism disorders (43). An increase in *Pevotella* in the gut microbiota can reduce body weight and cholesterol levels (44). *Oscillospira* is associated with improved metabolic activity and reduced lipid accumulation (45), while *Coprococcus* produces butyrate and acetate, enhancing intestinal barrier function and reducing steatosis risk (46, 47). Notably, this study observed elevated *Lactobacillus* abundance in HFD-fed mice, which was reduced after treatment with UFTBS and FTBS, consistent with previous studies (48, 49). Although bacteria belonging to the genus Lactobacillus often exhibit probiotic characteristics, its beneficial effects are strain-dependent and context-dependent (50). *Lactobacillus reuteri*, for example, was positively associated with obesity in humans (51). The reduction in *Blautia*, a bacterium linked to hyperlipidemia and diabetes, further supports FTBS’s lipid-lowering effects (1, 52).

#### 4.2.2 Enhancement of SCFA production and metabolic pathways

SCFAs, such as acetate, propionate, and butyrate, are crucial microbial metabolites that regulate host metabolism (53). HFD reduced SCFA levels, consistent with previous studies (54, 55). FTBS intervention significantly increased SCFA levels, particularly acetate, propionate, and butyrate, which are known to improve insulin sensitivity, reduce inflammation, and promote weight loss (56–58). The increase in SCFAs may be attributed to the enrichment of SCFA-producing bacteria such as *Prevotella*, *Bacteroides*, and *Coprococcus*.

Non-targeted metabolomics revealed that FTBS restored 192 serum metabolites to near-normal levels, primarily involved in valine, leucine, and isoleucine biosynthesis; glycerophospholipid metabolism; the citrate cycle; and primary bile acid biosynthesis. Branched-chain amino acids (BCAAs) play a critical role in lipid metabolism, and FTBS downregulated leucine biosynthesis while upregulating isoleucine and valine pathways, contributing to its lipid-lowering effects (22). Downregulation of glycerophospholipid metabolism, a key driver of lipid disorders, further supports FTBS’s therapeutic potential. The upregulation of the citrate cycle and primary bile acid biosynthesis by FTBS highlights its role in energy metabolism and cholesterol homeostasis. In this study, we found that oxoglutaric acid was significantly upregulated in the citrate cycle after treatment with FTBS. Oxoglutaric acid, a key intermediate in the citrate cycle, provides energy and exerts antioxidative effects (59). Enhanced bile acid synthesis promotes fat and cholesterol excretion, reducing serum and hepatic lipid levels (60, 61).

#### 4.2.3 Gut microbiota, SCFAs, and hepatic lipid metabolism

The interplay between gut microbiota, SCFAs, and hepatic lipid metabolism is a critical aspect of FTBS’s lipid-lowering effects. SCFAs, particularly butyrate and propionate, play a pivotal role in regulating hepatic lipid metabolism. Butyrate, produced by bacteria such as *Coprococcus* and *Oscillospira*, can activate AMPK signaling in the liver, leading to the inhibition of lipogenesis and promotion of fatty acid oxidation (62). Propionate, on the other hand, has been shown to reduce hepatic cholesterol synthesis by inhibiting HMG-CoA reductase, a key enzyme in the cholesterol biosynthesis pathway (63).

Additionally, SCFAs can modulate bile acid metabolism by activating the farnesoid X receptor (FXR) and G protein-coupled receptors (GPCRs) in the liver and intestine (64, 65). This activation enhances bile acid synthesis and excretion, thereby reducing hepatic lipid accumulation (60). The enrichment of SCFA-producing bacteria by FTBS, combined with the observed upregulation of primary bile acid biosynthesis, suggests a synergistic mechanism by which FTBS improves hepatic lipid metabolism.

Furthermore, the reduction in the F/B ratio and the increase in beneficial bacteria such as *Prevotella* and *Bacteroides* may contribute to the attenuation of hepatic steatosis. These bacteria are known to produce anti-inflammatory metabolites and enhance intestinal barrier function, reducing the translocation of lipopolysaccharides (LPS) and subsequent hepatic inflammation, a key driver of lipid accumulation (66).

### 4.3 Liver health improvement mechanisms: role of gut microbiota and SCFAs

The improvement in liver health observed with FTBS intervention can be attributed to the intricate relationship between gut microbiota, SCFAs, and hepatic lipid metabolism. The gut-liver axis plays a crucial role in maintaining liver homeostasis, and dysbiosis of the gut microbiota can lead to hepatic lipid accumulation and inflammation (67). FTBS’s ability to modulate gut microbiota composition and enhance SCFA production directly impacts liver health by improving lipid metabolism and reducing oxidative stress.

SCFAs, particularly butyrate, have been shown to improve liver morphology by reducing hepatic lipid accumulation and inflammation. Butyrate enhances the expression of genes involved in fatty acid oxidation and inhibits lipogenesis in the liver (62). Additionally, butyrate has anti-inflammatory properties that help mitigate liver inflammation, a common feature of non-alcoholic fatty liver disease (NAFLD) (68). Propionate, another key SCFA, contributes to liver health by modulating cholesterol metabolism. Propionate inhibits hepatic cholesterol synthesis by downregulating HMG-CoA reductase activity, thereby reducing serum and hepatic cholesterol levels (63). This mechanism is particularly relevant in the context of hyperlipidemia and NAFLD, where excessive cholesterol accumulation in the liver can exacerbate disease progression.

The integration of serum metabolomics data with gut microbiota and SCFA profiles provides further insights into the metabolic changes induced by FTBS. The upregulation of the citrate cycle and primary bile acid biosynthesis pathways, coupled with increased SCFA levels, suggests that FTBS enhances energy metabolism and cholesterol homeostasis in the liver. These metabolic changes are likely mediated by the enrichment of SCFA-producing bacteria, such as *Prevotella* and *Bacteroides*, which play a key role in maintaining gut-liver axis integrity.

In summary, FTBS ameliorates HFD-induced hyperlipidemia by modulating gut microbiota, enhancing SCFA production, and regulating key metabolic pathways. These findings highlight FTBS as a promising functional food for managing lipid metabolism disorders. Future studies should focus on identifying the specific bioactive compounds in FTBS and their mechanisms of action to further validate its therapeutic potential. Additionally, clinical trials are needed to confirm the efficacy and safety of FTBS in humans, particularly in individuals with early-stage hyperlipidemia or those at risk of developing metabolic syndrome. Further research could also explore the potential synergistic effects of FTBS with other dietary interventions or pharmacological treatments to optimize lipid-lowering strategies.

## 5 Conclusion

This study demonstrates that FTBS can effectively alleviate metabolic disorders induced by HFD, with superior efficacy compared to UFTBS. Comprehensive multi-omics analyses suggest that its protective effects are mediated through the modulation of the gut-liver axis. Specifically, FTBS remodels microbial ecosystem by enrichment of *Prevotella*, *Coprococcus*, and *Oscillospira*, which enhances SCFA production while suppressing obesogenic bacteria, restoring microbial diversity and F/B balance. Concurrently, systemic metabolic profiles were improved by restoration of 192 metabolites and significant reprogramming of core metabolic pathways such as branched-chain amino acid biosynthesis, primary bile acid biosynthesis, and citrate cycle. The synergistic improvements at the intestinal and systemic levels ultimately manifested as a significant alleviation of steatosis in liver tissue sections and a decrease in key blood lipid levels such as TC and TG. Our findings position FTBS as a promising nutraceutical for metabolic syndrome-related hepatic disorders. Prioritizing clinical validation and precise strain-function characterization will accelerate its translational application.

## Data Availability

The datasets generated for this study are available in the NCBI BioProject repository under accession number PRJNA1356646.

## Footnotes

1 http://www.hmdb.ca/; https://metlin.scripps.edu/

2
http://cloud.majorbio.com

3
https://www.kegg.jp/kegg/pathway.html
