# Supplementary material for: Serum metabolomics and gut microbiota analysis reveal the lipid-lowering effects of fermented triple-bean soup in high-fat diet-fed mice
Source: Front Nutr. 2025 Dec 3;12:1705483. doi: 10.3389/fnut.2025.1705483 (PMC12708937; doi:10.3389/fnut.2025.1705483)
Supplement: Supplementary file 1 [file Data_Sheet_1.docx]

**Table S1 Group information and administration.**

| Group | Treatment | |
| --- | --- | --- |
|  | 0ral administration | Intragastric administration |
| NFD | Standard diet+distilled water | 10 mL/ kg BW normal saline |
| HFD | High-fat diet+distilled water | 10 mL/ kg BW normal saline |
| UFTBS | High-fat diet +distilled water | 10 mL/ kg BW unfermented Triple-Bean Soup group |
| FTBS | High-fat diet+distilled water | 10 mL/ kg BW fermented Triple-Bean Soup group |

NFD, mice fed with a normal diet; HFD mice fed with an HFD; UFTBS, mice fed with an HFD supplemented with unfermented Triple-Bean Soup; FTBS, mice fed with an HFD supplemented with fermented Triple-Bean Soup.

**Table S2 Mass spectrum parameters**

| Description | parameter |
| --- | --- |
| Scan type（m/z） | 70-1050 |
| Sheath gas flow rate(arb) | 60 |
| Aux gas flow rate (arb) | 20 |
| Heater temp (℃) | 350 |
| Capillary temp (℃) | 320 |
| Spray voltage (+) (V) | 3400 |
| Spray voltage (-) (V) | -3000 |
| S-Lens RF Level | 70 |
| Normalized collision energy (eV) | 20,40,60 |
| Resolution (Full MS) | 60000 |
| Resolution (MS 2) | 15000 |

**TableS3. Metabolites in serum of mice that significantly improved by FTBS.**

| Metabolite | M/Z | RT(min) | Adducts | Formula | Library ID | HMDB Class | Levels |
| --- | --- | --- | --- | --- | --- | --- | --- |
| Panthenol | 206.1385 | 3.220567 | M+H | C9H19NO4 | HMDB0304820;HMDB0004231;MJDBOTE0000360 | Fatty Acyls | ↓ |
| 9,10-Dihydroxystearic acid | 334.2946 | 6.0779 | M+NH4 | C18H36O4 | LMFA02000142;HMDB0303981;HMDB0302281 | Fatty Acyls | ↓ |
| 3-Hydroxy-L-proline | 307.1146 | 0.64535 | 2M+FA-H | C5H9NO3 | HMDB0002113 | Carboxylic acids and derivatives | ↓ |
| PE(O-20:0/0:0) | 518.3551 | 7.001183 | M+H, M+Na | C25H54NO6P | LMGP02060005 | - | ↓ |
| 2-Isopropylmalic acid | 221.0666 | 0.807567 | M+FA-H | C7H12O5 | HMDB0000402 | Fatty Acyls | ↓ |
| 3-Isopropylmalic acid | 221.0667 | 1.604917 | M+FA-H | C7H12O5 | HMDB0012156 | Fatty Acyls | ↓ |
| Dihydroxyphenylacetic acid sulfate | 246.9918 | 3.647267 | M-H | C8H8O7S | HMDB0304904 | Not Available | ↓ |
| 1-Aminocyclopropanecarboxylic acid | 302.1358 | 3.806833 | 3M-H | C4H7NO2 | HMDB0036458 | Carboxylic acids and derivatives | ↓ |
| Zanamivir | 369.0826 | 3.913433 | M+K-2H | C12H20N4O7 | HMDB0014698 | Carboxylic acids and derivatives | ↓ |
| Solamargine | 912.4911 | 4.260117 | M+FA-H | C45H73NO15 | HMDB0258362;MJDBOTE0001240 | Azaspirodecane derivatives | ↓ |
| Safranal | 195.1027 | 5.223833 | M+FA-H | C10H14O | HMDB0036061 | Organic oxides | ↓ |
| Valtrate | 233.0431 | 5.388683 | M+Na-2H | C10H12O5 | HMDB0034493 | Prenol lipids | ↓ |
| 4-Allylpyrocatechol sulfate | 229.0176 | 5.954483 | M-H | C9H10O5S | HMDB0304934 | Organic sulfuric acids and derivatives | ↓ |
| 17beta-Estradiol 3-sulfate | 387.1018 | 5.975883 | M+Cl | C18H24O5S | HMDB0004448 | Steroids and steroid derivatives | ↓ |
| Quinestrol | 409.2357 | 6.02915 | M+FA-H | C25H32O2 | LMST02010037;HMDB0015579 | Steroids and steroid derivatives | ↓ |
| Cepanone | 209.1547 | 6.23665 | M-H | C13H22O2 | HMDB0032098 | Dihydrofurans | ↓ |
| (1E,4Z,6a,8b,10a)-8-(2-Methylbutanoyloxy)-10,15-dihydroxy-3-oxo-1,4,11(13)-germacratrien-12,6-olide | 377.1579 | 6.23665 | M-H | C20H26O7 | HMDB0041002 | Prenol lipids | ↓ |
| Milbemycin beta2 | 603.3539 | 6.24195 | M+FA-H | C33H50O7 | HMDB0254728;HMDB0254730;HMDB0254721;HMDB0242623 | Macrolides and analogues | ↓ |
| R-limonene | 413.218 | 5.959833 | M-H, 2M+FA-H | C10H16O3 | HMDB0003375;HMDB0257077 | Prenol lipids | ↓ |
| N-Arachidonoyl Methionine | 434.2732 | 6.549533 | M-H | C25H41NO3S | HMDB0242000 | Not Available | ↓ |
| Auberganol | 539.4315 | 6.6714 | 2M+Hac-H | C15H28O2 | HMDB0039715 | Prenol lipids | ↓ |
| DG(8:0/0:0/i-19:0) | 519.4055 | 6.698117 | M+Na-2H | C30H58O5 | HMDB0092940 | Glycerolipids | ↓ |
| Geosmin | 409.3322 | 6.703417 | 2M+FA-H | C12H22O | HMDB0036461 | Organooxygen compounds | ↓ |
| 18-hydroxyoleate | 593.4783 | 6.756417 | 2M-H | C18H33O3- | HMDB0304033 | Fatty Acyls | ↓ |
| DG(10:0/i-14:0/0:0) | 437.3637 | 6.842217 | M-H2O-H | C27H52O5 | HMDB0092975;PW_C064344 | Glycerolipids | ↓ |
| Octahydrohyperforin | 529.4263 | 6.927317 | M-H | C34H58O4 | HMDB0030463;HMDB0255901 | Prenol lipids | ↓ |
| Embelin | 293.1759 | 6.24725 | M-H, 2M-H | C17H26O4 | HMDB0251767;LMPK15050006 | Organooxygen compounds | ↓ |
| Cortisol 21-mesylate | 461.1589 | 7.331983 | M+Na-2H | C22H32O7S | HMDB0250467 | Steroids and steroid derivatives | ↓ |
| Curculigoside | 447.1342 | 7.315917 | M-H2O-H | C22H26O11 | HMDB0250592 | Organooxygen compounds | ↓ |
| 9-Hydroxylinoleic acid | 591.4626 | 6.703417 | M-H, 2M-H | C18H32O3 | HMDB0247599;HMDB0004670;HMDB0062652 | Fatty Acyls | ↓ |
| Glycerophospho-N-Oleoyl Ethanolamine | 478.2937 | 7.02835 | M-H | C23H46NO7P | - | - | ↓ |
| Cedrol | 503.4105 | 7.012367 | 2M+Hac-H | C15H26O | HMDB0303103;LMPR0103690007;HMDB0303492 | Prenol lipids | ↓ |
| N(4)-Octadecyl-1-arabinofuranosylcytosine | 494.3613 | 7.007033 | M-H | C27H49N3O5 | HMDB0248147 | Pyrimidine nucleosides | ↓ |
| 3a,7a-Dihydroxy-5b-cholestane | 449.3634 | 6.836917 | M+FA-H | C27H48O2 | PW_C002995;HMDB0006893 | Steroids and steroid derivatives | ↓ |
| 4-(undecan-5-yl)benzene-1-Sulfonic Acid | 311.1685 | 6.826233 | M-H | C17H28O3S | - | - | ↓ |
| Isovaleryl-Val-Val-Sta-OEt | 466.3299 | 6.804267 | M-H2O-H | C25H47N3O6 | HMDB0253705 | Peptidomimetics | ↓ |
| (3beta,5alpha,6beta,24R)-Stigmastane-3,5,6-triol | 493.3899 | 6.719317 | M+FA-H | C29H52O3 | LMST01040240;HMDB0030014 | Steroids and steroid derivatives | ↓ |
| DG(18:1(12Z)-2OH(9,10)/0:0/13:0) | 565.447 | 6.703417 | M-H2O-H | C34H64O7 | HMDB0294932 | Not Available | ↓ |
| N-(3-(Dimethylamino)propyl)acrylamide | 467.3739 | 6.703417 | 3M-H | C8H16N2O | HMDB0246231 | Carboxylic acids and derivatives | ↓ |
| Soyasapogenol D | 517.3897 | 6.698117 | M+FA-H | C31H52O3 | HMDB0034507 | Prenol lipids | ↓ |
| 5-Nonadecyl-1,3-benzenediol | 421.3322 | 6.698117 | M+FA-H | C25H44O2 | HMDB0030956 | Phenols | ↓ |
| 2,3-Dihydro-5-hydroxy-2,2-dipentyl-4,6-di-tert-butylbenzofuran | 433.3323 | 6.682233 | M+FA-H | C26H44O2 | HMDB0249329 | Coumarans | ↓ |
| N-Oleoyldopamine | 438.2988 | 6.629 | M+Na-2H | C26H43NO3 | HMDB0255218 | Phenols | ↓ |
| 3-(3,5-Di-tert-butyl-4-hydroxyphenyl)propionic acid | 277.1809 | 6.4115 | M-H | C17H26O3 | HMDB0245577 | Phenylpropanoic acids | ↓ |
| Dibutyl Phthalate | 277.1444 | 6.347983 | M-H | C16H22O4 | HMDB0033244;MJDBOTE0001756 | Benzene and substituted derivatives | ↓ |
| Alantolactone | 277.1445 | 6.2791 | M+FA-H | C15H20O2 | LMPR0103190013;HMDB0035906;MJDBOTE0001021 | Prenol lipids | ↓ |
| Aldosterone | 405.1893 | 6.24725 | M+FA-H | C21H28O5 | LMST02030026;HMDB0000037;PW_C000026 | Steroids and steroid derivatives | ↓ |
| Heliangin | 361.1631 | 6.24725 | M-H | C20H26O6 | HMDB0036692 | Fatty Acyls | ↓ |
| N'-nitrosoanabasine | 236.1053 | 6.24725 | M+FA-H | C10H13N3O | HMDB0041939 | Not Available | ↓ |
| Risbitin | 221.1548 | 6.24725 | M-H | C14H22O2 | HMDB0302980 | Organooxygen compounds | ↓ |
| 3,4,5-Trihydroxypentanoylcarnitine | 294.1543 | 0.816267 | M+H-H2O, M+H | C12H23NO7 | HMDB0241652 | Fatty Acyls | ↓ |
| 12-Oxo-2,3-dinor-10,15-phytodienoic acid | 309.1704 | 6.23665 | M+FA-H | C16H24O3 | HMDB0032090 | Fatty Acyls | ↓ |
| N-Ethyl trans-2-cis-6-nonadienamide | 226.1448 | 6.2207 | M+FA-H | C11H19NO | LMFA08020193;HMDB0032273 | Fatty Acyls | ↓ |
| Tetranor-12(R)-HETE | 265.1809 | 6.19945 | M-H | C16H26O3 | - | - | ↓ |
| O-Demethylmetoprolol | 298.1651 | 6.183567 | M+FA-H | C14H23NO3 | HMDB0255850 | Phenols | ↓ |
| 11-Dodecenoic acid | 593.4786 | 6.804267 | 2M-H, 3M-H | C12H22O2 | HMDB0032248;LMFA01030043 | Fatty Acyls | ↓ |
| (R)-2-(4-(Tert-butoxycarbonyl)morpholin-3-yl)acetic acid | 266.1009 | 6.039867 | M+Na-2H | C11H19NO5 | HMDB0247375 | Oxazinanes | ↓ |
| Tricin | 329.0668 | 6.0345 | M-H | C17H14O7 | LMPK12110873 | - | ↓ |
| 5-O-Methylembelin | 345.1455 | 6.02915 | M+K-2H | C18H28O4 | HMDB0040867;LMPK15050007 | Organooxygen compounds | ↓ |
| 3-[4-(sulfooxy)phenyl]propanoic acid | 245.0125 | 5.933117 | M-H | C9H10O6S | HMDB0125171 | Organic sulfuric acids and derivatives | ↓ |
| O-Desmethyl-lacosamide | 281.1143 | 5.62275 | M+FA-H | C12H16N2O3 | HMDB0060855 | Carboxylic acids and derivatives | ↓ |
| Cogoxin | 761.4049 | 5.340867 | M-H2O-H | C41H64O14 | HMDB0250399;HMDB0001917 | Steroids and steroid derivatives | ↓ |
| Prenyl glucoside | 269.1007 | 5.143867 | M+Na-2H | C11H20O6 | HMDB0031876 | Fatty Acyls | ↓ |
| Nifekalant | 404.1924 | 4.946967 | M-H | C19H27N5O5 | HMDB0255595 | Benzene and substituted derivatives | ↓ |
| LHRH, N-ac-Naphthyl(1)-(4-Cl-phe)(2)-trp(3)-arg(6)-ala(10)- | 732.337 | 4.90965 | M-2H | C73H95ClN18O13 | HMDB0254075 | Carboxylic acids and derivatives | ↓ |
| Morphine-6-glucuronide | 460.1611 | 4.754833 | M-H | C23H27NO9 | HMDB0041937;PW_C022690 | Morphinans | ↓ |
| Queuine | 295.1496 | 0.5128 | M+NH4 | C12H15N5O3 | HMDB0001495 | Pyrrolopyrimidines | ↓ |
| 1,11-Undecanedicarboxylic acid | 265.1443 | 6.2207 | M-H, M+Na-2H | C13H24O4 | HMDB0002327 | Fatty Acyls | ↓ |
| Caffeic acid 4-O-sulfate | 258.9918 | 4.355617 | M-H | C9H8O7S | HMDB0041708 | Cinnamic acids and derivatives | ↓ |
| 3-hydroxy-3-(3-hydroxyphenyl)propanoic acid-O-sulphate | 242.9969 | 4.307883 | M-H2O-H | C9H10O7S | HMDB0059967 | Hydroxy acids and derivatives | ↓ |
| 3-(3,5-dihydroxyphenyl)-1-propanoic acid sulphate | 242.9969 | 4.046717 | M-H2O-H | C9H10O7S | HMDB0061117 | Phenols | ↓ |
| Melilotoside | 371.0984 | 3.929667 | M+FA-H | C15H18O8 | HMDB0033581;HMDB0303353 | Organooxygen compounds | ↓ |
| Cyanidin 3-(caffeoyl-sophoroside) 5-glucoside | 466.6188 | 3.801517 | M-2H | C42H47O24+ | HMDB0302688 | Flavonoids | ↓ |
| Malvidin 3-rhamnoside 5-glucoside | 676.1412 | 3.3757 | M+K-2H | C29H35O16+ | HMDB0303640 | Flavonoids | ↓ |
| Ethyl Glucuronide | 221.0667 | 0.712417 | M-H | C8H14O7 | HMDB0010325 | Organooxygen compounds | ↓ |
| Lichenin | 207.051 | 0.6666 | M+FA-H | C6H10O5 | HMDB0302349 | Organooxygen compounds | ↓ |
| Miserotoxin | 309.1289 | 0.646017 | M+ACN+H | C9H17NO8 | HMDB0254754 | Organooxygen compounds | ↓ |
| 27-Hydroxycholesterol | 447.3472 | 6.80955 | M+FA-H | C27H46O2 | PW_C001414;HMDB0002103 | Steroids and steroid derivatives | ↓ |
| Glycerol 3-Phosphate | 171.0064 | 0.656017 | M-H | C3H9O6P | HMDB0252849;PW_C000081;HMDB0000126 | Glycerophospholipids | ↓ |
| Glycolic Acid | 75.00874 | 0.650667 | M-H | C2H4O3 | HMDB0000115;HMDB0304424 | Hydroxy acids and derivatives | ↓ |
| P-Coumaric Acid | 163.0401 | 5.372733 | M-H | C9H8O3 | HMDB0030677;HMDB0003654;HMDB0002035;MJDBOTE0000719 | Cinnamic acids and derivatives | ↓ |
| Fructosyl valine | 280.1387 | 0.7204 | M+H | C11H21NO7 | HMDB0252496 | Carboxylic acids and derivatives | ↓ |
| Cis-and trans-Ethyl 2,4-dimethyl-1,3-dioxolane-2-acetate | 206.1385 | 3.4341 | M+NH4 | C9H16O4 | - | - | ↓ |
| PC(DiMe(9,3)/PGF2alpha) | 457.7558 | 4.1448 | M+2Na | C46H80NO12P | HMDB0289247 | Not Available | ↓ |
| PC(PGF2alpha/DiMe(9,3)) | 457.7558 | 4.262033 | M+2Na | C46H80NO12P | HMDB0289248 | Not Available | ↓ |
| 8-Hydroxyquinoline | 146.0599 | 4.905983 | M+H | C9H7NO | - | - | ↓ |
| Smilagenin 3-[2''-glucosyl-6''-arabinosylglucoside] | 459.2288 | 5.4917 | M+2Na | C44H72O17 | HMDB0034309 | Anthracenes | ↓ |
| 8,8-Diethoxy-2,6-dimethyl-2-octanol | 288.2529 | 5.8118 | M+ACN+H | C14H30O3 | HMDB0034557 | Organooxygen compounds | ↓ |
| Hexanediol | 278.2322 | 5.923633 | 2M+ACN+H | C6H14O2 | HMDB0253145 | Fatty Acyls | ↓ |
| (2R,3R,4R,5S)-2-(Hydroxymethyl)-1-nonylpiperidine-3,4,5-triol | 322.2582 | 5.923633 | M+CH3OH+H | C15H31NO4 | HMDB0242572 | Piperidines | ↓ |
| Austalide B | 513.1858 | 5.934483 | M+K | C26H34O8 | HMDB0030004 | Organooxygen compounds | ↓ |
| 3-(L-Menthoxy)-2-methylpropane-1,2-diol | 262.2373 | 6.024767 | M+NH4 | C14H28O3 | HMDB0032417 | Prenol lipids | ↓ |
| PE(O-18:0/0:0) | 490.3258 | 6.80505 | M+Na | C23H50NO6P | LMGP02060003 | - | ↓ |
| 1,25-Dihydroxy-19-norvitamin D3 | 468.344 | 6.799767 | M+ACN+Na | C26H44O3 | HMDB0244153 | Steroids and steroid derivatives | ↓ |
| DHAP(O-18:0) | 440.3126 | 6.623333 | M+NH4 | C21H43O6P | HMDB0011142 | Organooxygen compounds | ↓ |
| Polypropylene | 372.3465 | 6.088467 | M+NH4 | C22H42O3 | HMDB0255988 | Fatty Acyls | ↓ |
| N-Dodecylsarcosinate | 290.2686 | 6.083167 | M+CH3OH+H | C15H31NO2 | HMDB0255129 | Carboxylic acids and derivatives | ↓ |
| MG(a-13:0/0:0/0:0)[rac] | 306.2633 | 6.014133 | M+NH4 | C16H32O4 | HMDB0072841 | Glycerolipids | ↓ |
| Acyline | 777.8663 | 4.87945 | M+H+Na, M+2Na | C80H102ClN15O14 | HMDB0247994 | Polypeptides | ↓ |
| PA(24:1(15Z)/20:3(8Z,11Z,14Z)-2OH(5,6)) | 443.2807 | 5.865233 | M+2Na | C47H85O10P | HMDB0266423 | Not Available | ↓ |
| Methyl ethaneperoxoate | 203.0524 | 0.624767 | M+ACN+H, 2M+Na | C3H6O3 | HMDB0244711 | Carboxylic acids and derivatives | ↓ |
| Zierin | 312.1107 | 0.826933 | M+H | C14H17NO7 | HMDB0301769 | Organooxygen compounds | ↓ |
| PC(TXB2/22:4(7Z,10Z,13Z,16Z)) | 473.7927 | 5.1933 | M+H+Na, M+2Na | C50H86NO12P | HMDB0288217 | Not Available | ↓ |
| Trihexosylceramide (d18:1/12:0) | 506.7967 | 5.246517 | M+H+Na, M+2Na | C48H89NO18 | HMDB0004877 | Sphingolipids | ↓ |
| PS(20:5(5Z,8Z,10E,14Z,17Z)-OH(12)/14:0) | 808.4185 | 5.763883 | M+K | C40H68NO11P | HMDB0280864 | Not Available | ↓ |
| Phenobarbital | 233.092 | 5.46495 | M+H-H2O, M+H | C12H12N2O3 | HMDB0015305 | Diazines | ↓ |
| Neuromedin B | 577.7638 | 4.831617 | M+H+Na | C52H73N15O12S | HMDB0013018 | Carboxylic acids and derivatives | ↓ |
| PE(16:1(9Z)/22:6(4Z,8Z,10Z,13Z,16Z,19Z)-OH(7)) | 400.7468 | 4.778517 | M+H+Na | C43H72NO9P | HMDB0261064 | Not Available | ↓ |
| PA(20:3(8Z,11Z,14Z)/20:5(7Z,9Z,11E,13E,17Z)-3OH(5,6,15)) | 408.2237 | 4.267333 | M+H+Na | C43H69O11P | HMDB0265101 | Not Available | ↓ |
| Icaridin | 262.201 | 4.123517 | M+CH3OH+H | C12H23NO3 | HMDB0253340 | Piperidines | ↓ |
| Dermorphin | 413.1843 | 3.488133 | M+H+Na | C40H50N8O10 | HMDB0251025 | Carboxylic acids and derivatives | ↓ |
| Pro-Pro-Pro | 310.1757 | 2.091017 | M+H | C15H23N3O4 | HMDB0256689 | Carboxylic acids and derivatives | ↑ |
| PE(18:1(9Z)/0:0) | 480.3084 | 6.549 | M+H, M+Na | C23H46NO7P | LMGP02050004 | - | ↑ |
| 2-Amino-4-[carbamimidoyl(methyl)amino]butanoic acid | 173.1044 | 0.613333 | M-H | C6H14N4O2 | HMDB0255374 | Carboxylic acids and derivatives | ↑ |
| Oxoglutaric acid | 145.0143 | 0.800617 | M-H | C5H6O5 | PW_C000134;HMDB0000208 | Keto acids and derivatives | ↑ |
| Elenolide | 269.0667 | 3.817433 | M+FA-H | C11H12O5 | HMDB0030018 | Pyrans | ↑ |
| Sagopilone | 580.218 | 3.892233 | M+K-2H | C30H41NO6S | HMDB0257445 | Macrolides and analogues | ↑ |
| Indoxyl glucuronide | 308.0776 | 3.902817 | M-H | C14H15NO7 | HMDB0010319 | Organooxygen compounds | ↑ |
| N-lactoyl-Tryptophan | 275.1038 | 5.707967 | M-H | C14H16N2O4 | HMDB0062178 | Carboxylic acids and derivatives | ↑ |
| Cephaeline | 501.2565 | 5.9169 | M+Cl | C28H38N2O4 | HMDB0249806 | Emetine alkaloids | ↑ |
| S-(PGA1)-glutathione | 642.3066 | 5.975883 | M-H | C30H49N3O10S | HMDB0013061 | Carboxylic acids and derivatives | ↑ |
| DG(8:0/8:0/0:0) | 389.2547 | 6.045233 | M+FA-H | C19H36O5 | PW_C062137;HMDB0116368 | Glycerolipids | ↑ |
| 20-Hydroxyeicosatetraenoic acid | 365.2332 | 6.141083 | M+FA-H | C20H32O3 | HMDB0005998;PW_C002614 | Fatty Acyls | ↑ |
| DG(20:5(5Z,8Z,11Z,14Z,16E)-OH(18R)/8:0/0:0) | 563.357 | 6.226017 | M+FA-H | C31H50O6 | HMDB0297184 | Not Available | ↑ |
| Prostaglandin E1 | 353.2331 | 6.231317 | M-H | C20H34O5 | LMFA03010134;HMDB0302206;HMDB0001442 | Fatty Acyls | ↑ |
| (5Z,8Z,10E,12S,14Z)-12-Hydroperoxyicosa-5,8,10,14-tetraenoylcarnitine | 516.2728 | 6.24725 | M+K-2H | C27H45NO6 | HMDB0241589 | Fatty Acyls | ↑ |
| Arctiol | 281.2122 | 6.342717 | M+FA-H | C16H28O | HMDB0301955 | Prenol lipids | ↑ |
| LysoPE(0:0/20:5(5Z,8Z,11Z,14Z,17Z)) | 498.2631 | 6.358583 | M-H | C25H42NO7P | HMDB0011489 | Glycerophospholipids | ↑ |
| PE(22:6/0:0) | 524.2779 | 6.39565 | M-H | C27H44NO7P | - | - | ↑ |
| Glycocholic acid | 500.278 | 6.427383 | M+Cl | C26H43NO6 | HMDB0000331;LMST05030001;HMDB0000138;PW_C000089 | Steroids and steroid derivatives | ↑ |
| 20,26-Dihydroxyecdysone | 531.2729 | 6.475033 | M+Cl | C27H44O8 | LMST01010184;HMDB0245570 | Steroids and steroid derivatives | ↑ |
| PE(20:3/0:0) | 502.2937 | 6.480333 | M-H | C25H46NO7P | - | - | ↑ |
| Ponasterone A | 973.6262 | 6.53895 | 2M+FA-H | C27H44O6 | HMDB0256697;LMST01010195 | Steroids and steroid derivatives | ↑ |
| PE(18:1/0:0) | 478.2936 | 6.549533 | M-H | C23H46NO7P | LMGP02050004 | - | ↑ |
| Cer(d20:1/20:3(5Z,11Z,14Z)-O(8,9)) | 628.5306 | 6.6555 | M-H | C40H71NO4 | HMDB0290097 | Not Available | ↑ |
| Resolvin D2 | 421.2208 | 6.231317 | M+FA-H, 2M+FA-H | C22H32O5 | HMDB0002294;LMFA04030001 | Fatty Acyls | ↑ |
| GPCho(3:0/17:2) | 562.3491 | 6.2528 | M+H, M+Na | C28H52NO8P | - | - | ↑ |
| Beta-tocotrienol | 409.3108 | 6.948617 | M-H | C28H42O2 | LMPR02020055;HMDB0304597;HMDB0030554 | Prenol lipids | ↑ |
| Prostaglandin E2 methyl ester | 387.2152 | 6.7352 | M+Na-2H | C21H34O5 | HMDB0256853 | Fatty Acyls | ↑ |
| PE(20:2/0:0) | 504.3095 | 6.560133 | M-H | C25H48NO7P | - | - | ↑ |
| 1-Stearoylglycerophosphoserine | 546.281 | 6.549533 | M+Na-2H | C24H48NO9P | HMDB0061698 | Glycerophospholipids | ↑ |
| PC(22:1(13Z)/LTE4) | 1019.61 | 6.432667 | M+Na-2H | C53H95N2O11PS | HMDB0287901 | Not Available | ↑ |
| LysoPE(20:4(8Z,11Z,14Z,17Z)/0:0) | 522.2623 | 6.342717 | M+Na-2H | C25H44NO7P | HMDB0011518 | Glycerophospholipids | ↑ |
| (11R,16S)-misoprostol | 381.2644 | 6.2791 | M-H | C22H38O5 | HMDB0242321 | Fatty Acyls | ↑ |
| 13,14-Dihydro PGE1 | 355.249 | 6.24195 | M-H | C20H36O5 | HMDB0002689 | Fatty Acyls | ↑ |
| Janthitrem C | 604.3254 | 6.231317 | M+Cl | C37H47NO4 | HMDB0040684 | Peptidomimetics | ↑ |
| KB 2 | 453.1584 | 6.226017 | M-H | C25H26O8 | HMDB0033666 | Flavonoids | ↑ |
| Calcitriol lactone | 489.2856 | 6.215367 | M+FA-H | C27H40O5 | HMDB0000969;HMDB0249548 | Steroids and steroid derivatives | ↑ |
| Leontogenin | 491.3013 | 6.17825 | M+FA-H | C27H42O5 | HMDB0033583 | Organooxygen compounds | ↑ |
| Leukotriene C4 | 624.2959 | 6.119883 | M-H | C30H47N3O9S | HMDB0001198;LMFA03020003;HMDB0005095;PW_C000933 | Carboxylic acids and derivatives | ↑ |
| 4-Hydroxyretinoic acid | 361.2019 | 6.08255 | M+FA-H | C20H28O3 | PW_C002690;HMDB0006254 | Prenol lipids | ↑ |
| Imidazolone A | 353.1218 | 6.02385 | M+Cl | C12H22N4O6 | HMDB0253421 | Carboxylic acids and derivatives | ↑ |
| Penilloic acid | 343.0911 | 5.707967 | M+Cl | C15H20N2O3S | HMDB0256237 | Carboxylic acids and derivatives | ↑ |
| Antibiotic GR 95647X | 443.2473 | 7.007033 | M-H, M+Cl, M+FA-H | C26H36O6 | HMDB0033034 | Phenols | ↑ |
| 5-(4-Carboxybutylperoxy)pentanoic acid | 215.0924 | 5.010717 | M-H2O-H | C10H18O6 | HMDB0260291 | Carboxylic acids and derivatives | ↑ |
| Candoxatrilat | 436.1724 | 4.8459 | M+K-2H | C20H33NO7 | HMDB0249582 | Carboxylic acids and derivatives | ↑ |
| Blepharin | 308.0776 | 4.14815 | M-H2O-H | C14H17NO8 | HMDB0029344 | Organooxygen compounds | ↑ |
| Leukotriene E3 | 440.2476 | 6.183567 | M-H, M+Na-2H | C23H39NO5S | LMFA03020074;HMDB0002355 | Fatty Acyls | ↑ |
| Acolongifloriside K | 619.2583 | 3.737767 | M+Cl | C29H44O12 | HMDB0247956;HMDB0031448 | Steroids and steroid derivatives | ↑ |
| Val-Pro-Asp-Pro-Arg | 619.2589 | 3.615417 | M+K-2H | C25H42N8O8 | HMDB0244421 | Carboxylic acids and derivatives | ↑ |
| 2-(6-Hydroxy-3-oxo-3H-xanthen-9-yl)benzoic acid | 367.0372 | 3.503267 | M+Cl | C20H12O5 | HMDB0244936 | Benzopyrans | ↑ |
| Melatonin glucuronide | 443.124 | 3.4979 | M+Cl | C19H24N2O8 | HMDB0060830 | Nucleoside and nucleotide analogues | ↑ |
| Cascarillin | 389.1967 | 6.08255 | M-H2O-H, M+Na-2H | C22H32O7 | HMDB0036836 | Prenol lipids | ↑ |
| L-2-Amino-6-oxohexanoic acid | 190.0721 | 2.08665 | M+FA-H | C6H11NO3 | HMDB0303320 | Carboxylic acids and derivatives | ↑ |
| Oxidized Glutathione | 611.1443 | 0.6666 | M-H | C20H32N6O12S2 | PW_C001847;HMDB0011691;HMDB0003337;MJDBOTE0000407 | Carboxylic acids and derivatives | ↑ |
| D-Mannose 6-Phosphate | 259.0224 | 0.6613 | M-H | C6H13O9P | HMDB0001078;HMDB0304312 | Organooxygen compounds | ↑ |
| N(6)-Methyllysine | 193.1544 | 0.635317 | M+CH3OH+H | C7H16N2O2 | HMDB0002038 | Carboxylic acids and derivatives | ↑ |
| Betaine | 235.165 | 0.646017 | 2M+H | C5H11NO2 | PW_C000030;HMDB0000043;MJDBTMA0000064 | Carboxylic acids and derivatives | ↑ |
| Lysinoalanine | 275.1709 | 0.656667 | M+ACN+H | C9H19N3O4 | HMDB0029447 | Carboxylic acids and derivatives | ↑ |
| Etiocholanolone glucuronide | 465.2471 | 6.231317 | M-H | C25H38O8 | PW_C002144;HMDB0004484;HMDB0002829 | Steroids and steroid derivatives | ↑ |
| Forskolin | 391.2125 | 6.109283 | M-H2O-H | C22H34O7 | LMPR0104030004;HMDB0032064;HMDB0252457;MJDBOTE0001052 | Benzofurans | ↑ |
| Triethanolamine | 321.2015 | 0.6673 | 2M+Na | C6H15NO3 | HMDB0032538 | Organonitrogen compounds | ↑ |
| Oxiracetam | 123.0551 | 0.800333 | M+H-2H2O | C6H10N2O3 | HMDB0255986 | Carboxylic acids and derivatives | ↑ |
| Caffeoyl tyrosine | 308.0907 | 1.151383 | M+H-2H2O | C18H17NO6 | HMDB0029295 | Carboxylic acids and derivatives | ↑ |
| Niacinamide | 123.0552 | 1.209783 | M+H | C6H6N2O | HMDB0001406;PW_C001086 | Pyridines and derivatives | ↑ |
| Valeric acid | 144.1017 | 0.66195 | M+ACN+H | C5H10O2 | LMFA01010005;HMDB0000892 | Fatty Acyls | ↑ |
| (R)-2,3-Dihydroxy-3-methylvalerate | 190.1073 | 4.469567 | M+ACN+H | C6H12O4 | HMDB0012140 | Fatty Acyls | ↑ |
| [3-[2-Aminoethoxy(hydroxy)phosphoryl]oxy-2-hydroxypropyl] hexadecanoate | 454.292 | 6.543733 | M+H-H2O, M+H, M+Na | C21H44NO7P | HMDB0256030 | Glycerophospholipids | ↑ |
| Glutathione, oxidized | 307.0828 | 1.9369 | M+2H, M+H | C20H32N6O12S2 | HMDB0011691;HMDB0003337 | Carboxylic acids and derivatives | ↑ |
| 2'-O-Methylcytidine | 537.194 | 0.608717 | M+2Na-H, 2M+Na | C10H15N3O5 | HMDB0242132 | Pyrimidine nucleosides | ↑ |
| LysoPA(20:3(8Z,11Z,14Z)/0:0) | 502.2924 | 6.433217 | M+ACN+H | C23H41O7P | HMDB0114759 | Glycerophospholipids | ↑ |
| Penitrem E | 582.3157 | 6.236933 | M+H-H2O | C37H45NO6 | HMDB0256241;HMDB0242736 | Naphthopyrans | ↑ |
| LysoPE(22:5(7Z,10Z,13Z,16Z,19Z)/0:0) | 560.3336 | 6.236933 | M+CH3OH+H | C27H46NO7P | HMDB0011525 | Glycerophospholipids | ↑ |
| Hexadecenoylcarnitine | 420.3079 | 6.1524 | M+Na | C23H43NO4 | HMDB0253127 | Fatty Acyls | ↑ |
| Isofebrifugine | 644.3199 | 5.971683 | 2M+ACN+H | C16H19N3O3 | HMDB0242253;HMDB0253633 | Diazanaphthalenes | ↑ |
| 3b,8b-Dihydroxy-6b-(3-chloro-2-hydroxy-2-methylbutanoyloxy)-7(11)-eremophilen-12,8-olide | 434.1948 | 5.8812 | M+NH4 | C20H29ClO7 | HMDB0036524 | Prenol lipids | ↑ |
| Aspartame | 277.1178 | 5.705117 | M+H-H2O | C14H18N2O5 | HMDB0001894;MJDBOTE0001640 | Carboxylic acids and derivatives | ↑ |
| Xanthoxylin | 238.1071 | 5.64645 | M+ACN+H | C10H12O4 | HMDB0029645 | Organooxygen compounds | ↑ |
| Hydrocinnamic acid | 192.1017 | 5.64645 | M+ACN+H | C9H10O2 | HMDB0000764 | Phenylpropanoic acids | ↑ |
| Calanolide A | 434.1949 | 5.507667 | M+ACN+Na | C22H26O5 | HMDB0249535 | Coumarins and derivatives | ↑ |
| 2,3-dihydroxy-3-methylvalerate | 190.1072 | 4.320517 | M+ACN+H | C6H12O4 | HMDB0304038;LMFA01050469 | Fatty Acyls | ↑ |
| 6-Hydroxyhexadecanoylcarnitine | 398.3258 | 6.1524 | M+H-H2O, M+H | C23H45NO5 | HMDB0241468 | Fatty Acyls | ↑ |
| (8Z,11Z,14Z,17Z)-Icosa-8,11,14,17-tetraenoylcarnitine | 448.3413 | 6.157683 | M+H, M+Na | C27H45NO4 | HMDB0241588 | Fatty Acyls | ↑ |
| 10,11-Dihydroxycarbamazepine | 582.2318 | 3.873383 | 2M+ACN+H | C15H14N2O3 | PW_C040557;HMDB0060568 | Benzazepines | ↑ |
| Penitrem B | 584.3335 | 6.210483 | M+H, M+Na | C37H45NO5 | HMDB0256239;HMDB0242736 | Naphthopyrans | ↑ |
| Leucyl-leucine | 245.1857 | 3.364917 | M+H | C12H24N2O3 | HMDB0028933;HMDB0254039 | Carboxylic acids and derivatives | ↑ |


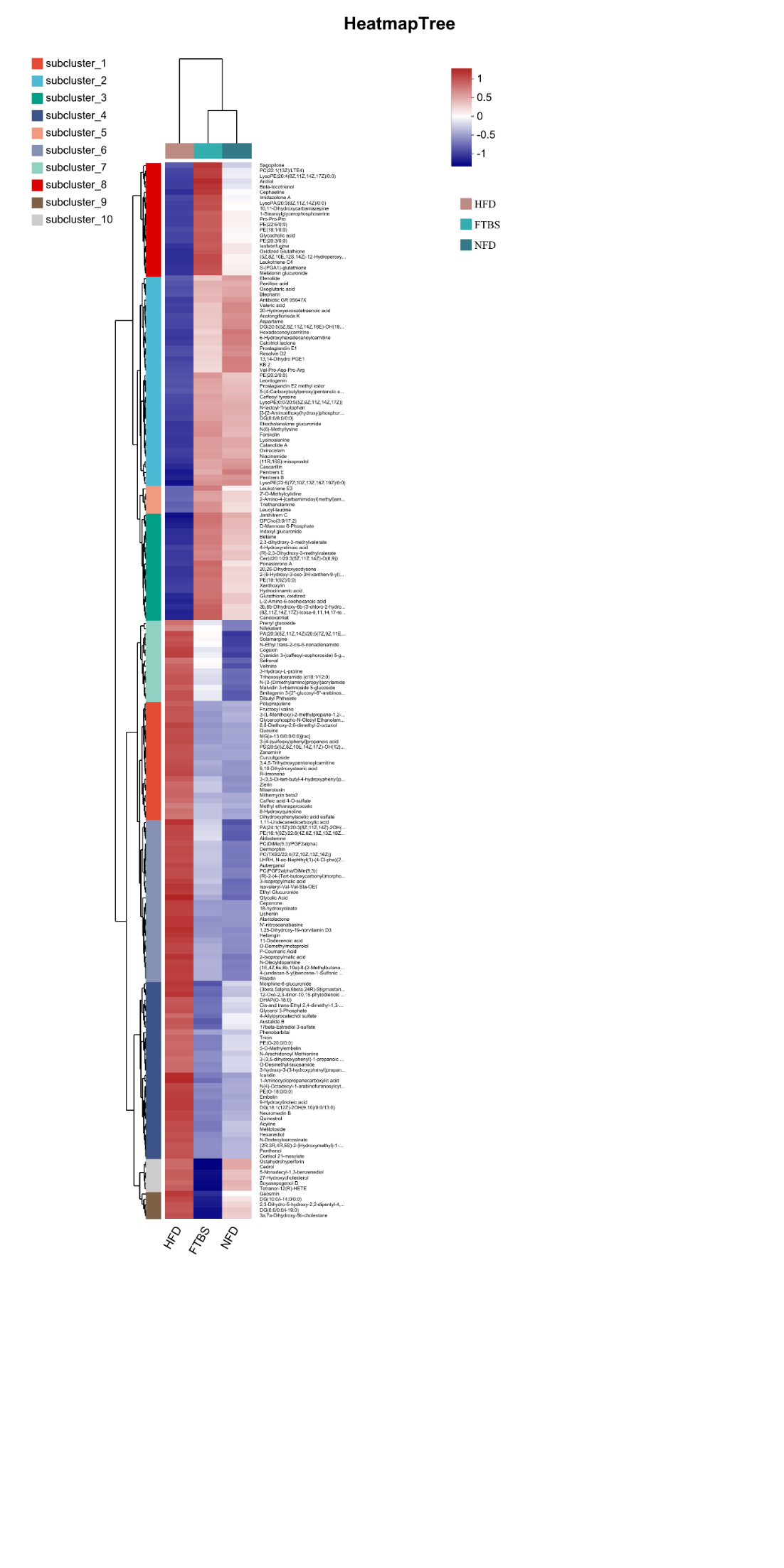
**Fig. S1** Heatmap of relative abundance of significant different metabolites in NFD, HFD and FTBS groups.
